# Supplementary material for: Patterns of Intron Gain and Loss in Fungi
Source: PLoS Biol. 2004 Nov 30;2(12):e422. doi: 10.1371/journal.pbio.0020422 (PMC532390; doi:10.1371/journal.pbio.0020422)
Supplement: Table S1 — Also available at http://genes.mit.edu/NielsenEtAl/. (4.3 MB ZIP). [file pbio.0020422.st001.zip › NielsenEtAl/html/283.html]

AN3058.1.NCU02274.1.MG00923.1.FG06290.1


```
 CLUSTAL W (1.82) Multiple Sequence Alignments - Introns Inserted


Sequence 1: AN3058.1	458 aa
Sequence 2: MG00923.1	468 aa
Sequence 3: FG06290.1	491 aa
Sequence 4: NCU02274.1	480 aa
Alignment Length: 491 aa
Number Identitical Residues: 326 aa
Alignment Score (without introns) 14511


MG00923.1 	----------------~MLEKSLLDSDPEVAEIM0KHEIQRQRESIILIASENVTSRAVF
NCU02274.1	----MSTYSLSETHKA0MLEHSLVESDPQVAEIM~KKEVQRQRESIILIASENVTSRAVF
FG06290.1 	MSPTNAEYALSESHKE0MLEKSLLDSDPEVASIM0KDEIQRQRESIVLIASENITSRAVF
AN3058.1  	----------------~-MEDRLVDTDPEVAKIM0ENEIQRQRESVVLIASENFTSRAVF
          	                  :*. *:::**:**.** :.*:******::******.******

MG00923.1 	DALGSPMSNKYSEGYPGKRYYGGNQHIDEIELLCQRRALAAFNVTEDKWGVNVQCLSGSP
NCU02274.1	DALGSPMSNKYSEGLPGARYYGGNQHIDEIEVLCQNRALEAFHLDPKQWGVNVQCLSGSP
FG06290.1 	DALGSPMSNKYSEGYPGARYYGGNQHIDQIELLCQRRALEAFHLDSEKWGVNVQCLSGSP
AN3058.1  	DALGSPMCNKYSEGYPGARYYGGNQHIDAIELLCQSRALKAFNLDADKWGVNVQCLSGSP
          	*******.****** ** ********** **:*** *** **::  .:************

MG00923.1 	ANLQVYQAIMPPHGRLMGLDLPHGGHLSHGYQTPQR-~------K2ISAVSTYFETMPYR
NCU02274.1	ANLQVYQAIMPVHGRLMGLDLPHGGHLSHGYQTPQR-~------K2ISAVSTYFETMPYR
FG06290.1 	ANLQVYQAIMPPHGRLMGLDLPHGGHLSHGYQTPARN2MLTIDNR~ISAVSTYFETMPYR
AN3058.1  	ANLQVYQALMRPHDRLMGLDLPHGGHLSHGYQTPSR-~------K2ISAVSTYFETFPYR
          	********:*  *.******************** *        : **********:***

MG00923.1 	VDLETGIIDYDQLEKNVVLYRPKILVAGTSAYCRLIDYARMRKIADLVGAYLVVDIAHIS
NCU02274.1	VNIDTGLIDYDTLEKNAQLFRPKVLVAGTSAYCRLIDYERMRKIADSVGAYLVVDMAHIS
FG06290.1 	VDLDTGIIDYDTLQKNAILYRPKVLVAGTSAYCRLIDYERMRKIADSVGAYLVVDMAHIS
AN3058.1  	VNLETGIIDYDTLEANAELYRPKILVAGTSAYCRLIDYARMRKIADKVGAYLVVDMAHIS
          	*:::**:**** *: *. *:***:************** ******* ********:****

MG00923.1 	GLVASGVIPSPFLHADVVTTTTHKSLRGPRGAMIFFRRGVRSVDPKTGKETMYDLEEPIN
NCU02274.1	GLIASEVIPSPFLYADVVTTTTHKSLRGPRGAMIFFRRGVRSVDAKTGKETLYDLEDKIN
FG06290.1 	GLIAAEVIPTPFKYADIVTTTTHKSLRGPRGAMIFFRKGVRSVDAKTGKETLYDLENPIN
AN3058.1  	GLIAAGVIPSPFEYADVVTTTTHKSLRGPRGAMIFFRKGVRSTDPKTGKDIMYDLEGPIN
          	**:*: ***:** :**:********************:****.*.****: :****  **

MG00923.1 	FSVFPGHQGGPHNHTITALAVALKQASTPEFKAYQQQVVDNAKALENSFKELGHKLVSDG
NCU02274.1	FSVFPGHQGGPHNHTITALAVALKQAASPEFKEYQQKVVANAKALEKKLKELGYKLVSDG
FG06290.1 	FSVFPGHQGGPHNHTITALAVALKQAASPDFKAYQEKVVSNAKTLENTFKALGHKLVSDG
AN3058.1  	FSVFPGHQGGPHNHTITALSVALKYAATTEFKQYQEQVIKNAKALENEFKAIGHKLVSDG
          	*******************:**** *::.:** **::*: ***:**: :* :*:******

MG00923.1 	TDSHMVLVDLRAHSLDGARVEAVLEQINIACNKNSIPGDKSALTPCGIRIGTPAMTSRGF
NCU02274.1	TDSHMVLVDLRPIGVDGARVEFLLEQINITCNKNAVPGDKSALTPGGLRIGTPAMTSRGF
FG06290.1 	TDSHMVLIDLRQHNLDGARVEAVLEQINIACNKNSIPGDKSALTPCGIRIGTPAMTSRGF
AN3058.1  	TDSHMVLVDLRPKSLDGARVEAVLEQINIACNKNSIPGDKSALTPCGIRIGAPAMTSRGM
          	*******:***  .:****** :******:****::********* *:***:*******:

MG00923.1 	GVEHFQRVAKYIDESIKICKDVQAALPKEANKLKDFKAKVASGEVSRINELKKEIASWCQ
NCU02274.1	GEADFEKVAVFVDEAVKLCKEIQASLPKEANKQKDFKAKIATSDIPRINELKQEIAAWSN
FG06290.1 	GEKEFERVGKFIDEAIKICKEEQAALPKEANKLKDFKARVASGEVQKINDFRKEIASWCS
AN3058.1  	GEEDFKRIARYIDQAINICKSVQAALPTDANKLKDFKAKVASGTVPEINDLRKEIAAWAS
          	*  .*:::. ::*:::::**. **:**.:*** *****::*:. : .**::::***:*..

MG00923.1 	TFPLPVEGWRTDAGI
NCU02274.1	TFPLPVEGWRYDAGL
FG06290.1 	AFPLPVEGWRLDASI
AN3058.1  	TFPLPV---------
          	:*****
```
